# Bladder Neoplasia in Pediatric Patients—A Single-Center Experience Including a Case Series

**DOI:** 10.3390/children10101596

**Published:** 2023-09-25

**Authors:** Frank-Martin Haecker, Elisabeth Bruder

**Affiliations:** 1 Department of Pediatric Surgery, Children’s Hospital of Eastern Switzerland, 9006 St. Gallen, Switzerland; 2 Faculty of Medicine, University of Basel, 4001 Basel, Switzerland; 3 Institute of Medical Genetics and Pathology, University Hospital Basel, 4031 Basel, Switzerland

**Keywords:** bladder neoplasia, bladder lesion, urothelial neoplasia, PUNLMP, rhabdomyosarcoma, children

## Abstract

**Objective:** Bladder lesions like urothelial carcinoma are rare in the first two decades of life. A biopsy of the bladder or urinary cytological examination is seldom required. Gross painless hematuria is the most relevant clinical syndrome. **Methods:** A retrospective analysis of surgical pathology records collected between 1984 and 2014 at our institution was performed in a search for cases of urothelial neoplasms originating within the urinary bladder in pediatric patients. Diagnoses were confirmed based on pathologic examination using the 2004 World Health Organization (WHO) classification system. We selected keywords such as bladder neoplasia, bladder lesion, urothelial neoplasia, rhabdomyosarcoma, and children. In addition, we describe clinical presentation and diagnostic procedures as well as treatment and follow-up of two patients. A review of the literature was performed to analyze recommendations concerning diagnostic staging, treatment, and follow-up examinations as well as surveillance of urothelial tumors in the pediatric population. **Results:** Screening the pathology database of the Institute of Medical Genetics and Pathology of the University Hospital Basel between 1988 and 2014 yielded 287 samples involving the urinary bladder, 110 autopsies, 135 biopsies, and 42 cytology specimens. Of these, most samples originated from malformations and inflammation. Only five were tumors: two were urothelial tumors and three were rhabdomyosarcomas. The majority of specimens comprised resections of the diverticula or distal ureter. Our case reports include two patients with a urothelial tumor. Among the urothelial tumors, one was a papillary urothelial neoplasm of low malignant potential (PUNLMP). Painless hematuria was the directing clinical symptom. The tumor was investigated by FISH, and a 9p21 deletion was found. The second tumor-like lesion was a fibroepithelial polyp arising from the bladder neck. **Conclusions:** Bladder tumors in children are rare and mostly consist of urothelial and mesenchymal neoplasms. Rhabdomyosarcoma is the most common malignant bladder tumor in childhood. Similar to adult urothelial neoplasms, the loss of 9p21 is also implicated in urothelial neoplasms in childhood. Despite an increasing number of case reports and small series published within the last 2 decades, general treatment protocols including recommendations for staging, tumor markers, and follow-up examinations are still not yet available for this tumor entity in the pediatric population.

## 1. Introduction

Bladder tumors, especially urothelial carcinoma, represent a rare entity in the first two decades of life. Under the age of 20, they are distinctly unusual with an incidence of 0.1 to 0.4%; most of them are described in case reports and small series [1,2,3,4,5,6,7,8,9,10,11,12,13,14,15,16]. Most of these series describe urothelial tumors as being characteristically superficial and low-grade. To evaluate the incidence of these lesions in a single center, we performed a retrospective study of our database comprising 26 years. In addition, we describe the clinical presentation, diagnostic work-up, treatment, and follow-up examinations of two patients.

## 2. Methods

A retrospective analysis of surgical pathology records collected between 1984 and 2014 at the Institute of Medical Genetic and Pathology of the University Hospital Basel was performed. We applied keywords such as bladder neoplasia, bladder lesion, urothelial neoplasia, rhabdomyosarcoma, and children. The age group “children” was defined from newborn to 16 years of age. We identified five pediatric patients with a tumor. Two out of five patients underwent transurethral resection of their tumor. Clinical presentation, applied diagnostic procedures, treatment, and follow-up of these two patients are described in detail.

A review of the recent literature was performed to analyze recommendations for diagnostic staging, treatment, and follow-up examinations as well as surveillance of patients suffering from these lesions in the pediatric population.

## 3. Results

We performed a retrospective analysis of the pathology database at the Institute of Medical Genetic and Pathology of the University Hospital Basel, considering samples collected between 1988 and 2014 (corresponding period comparable to other studies). We identified 287 samples involving the urinary bladder, 110 autopsies, 135 biopsies, and 42 cytology specimens. Most of these samples originated from (congenital) malformations and inflammation. The majority of specimens comprised resections of the diverticula or distal ureter. Only five were tumors: two were urothelial tumors and three were rhabdomyosarcomas.

Among the urothelial tumors, one was a papillary urothelial neoplasm of low malignant potential (PUNLMP). The tumor was investigated by FISH, and a 9p21 deletion was found (patient No. 1). The second one was a fibroepithelial polyp arising from the bladder neck (patient No. 2).

### 3.1. Patient No. 1

A 13-year-old boy presented with one episode of painless macrohematuria. The patient’s history before presentation was completely uneventful. The family’s history was negative for bladder neoplasia. There was no traveling to foreign countries and no exposition to well-known risk factors like cigarette smoking or occupational exposures such as aniline dyes, phenacetin, and low fluid consumption. Clinical examination demonstrated no significant abnormality, without any evidence of trauma or manipulation at the external genitalia. Blood tests including coagulation status as well as urinalysis including bacteriology revealed no pathological result.

**Figure 1:**
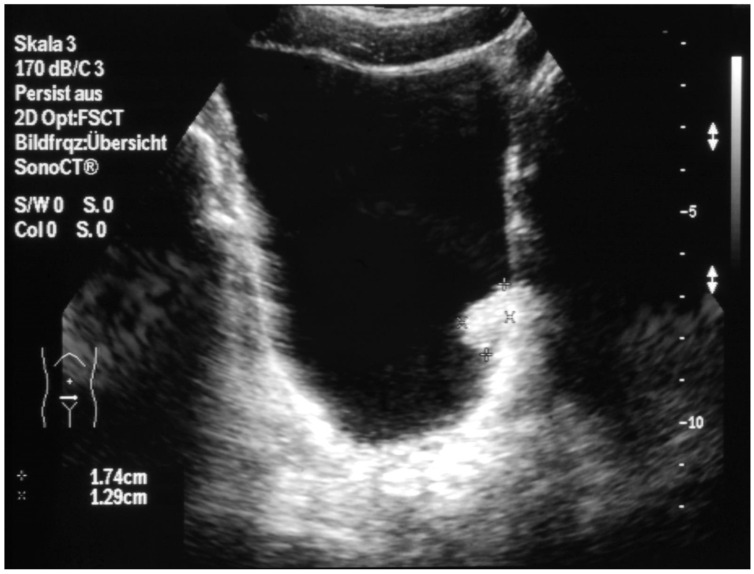
Papillary mass located on the lateral bladder wall close to the left ureteral ostium.

Abdominal ultrasound showed a papillary mass measuring 1.5 cm in diameter, located on the lateral bladder wall close to the left ureteral ostium (Figure 1). An abdominal CT scan demonstrated no evidence of additional tumor manifestations.

**Figure 2:**
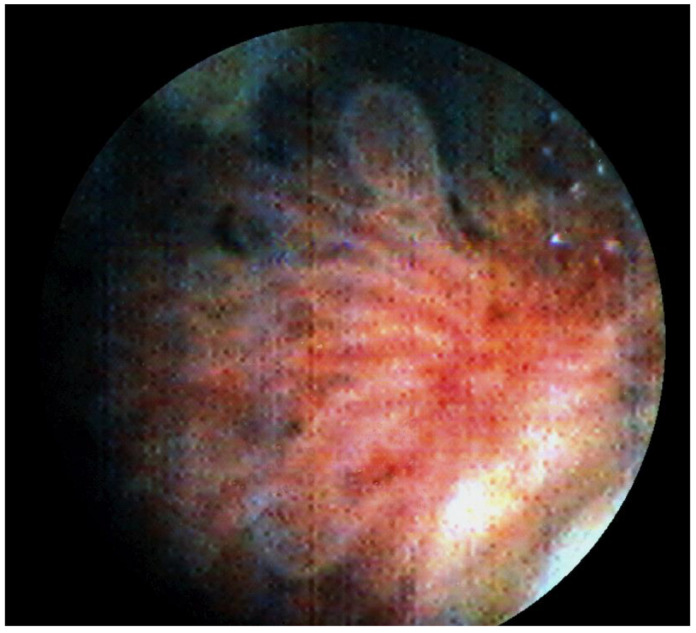
Cystoscopy showing a papillary tumor close to the left ureteral ostium.

We performed transurethral cystoscopy which confirmed a papillary tumor close to the left ureteral ostium (Figure 2). The tumor was excised by complete transurethral resection.

**Figure 3:**
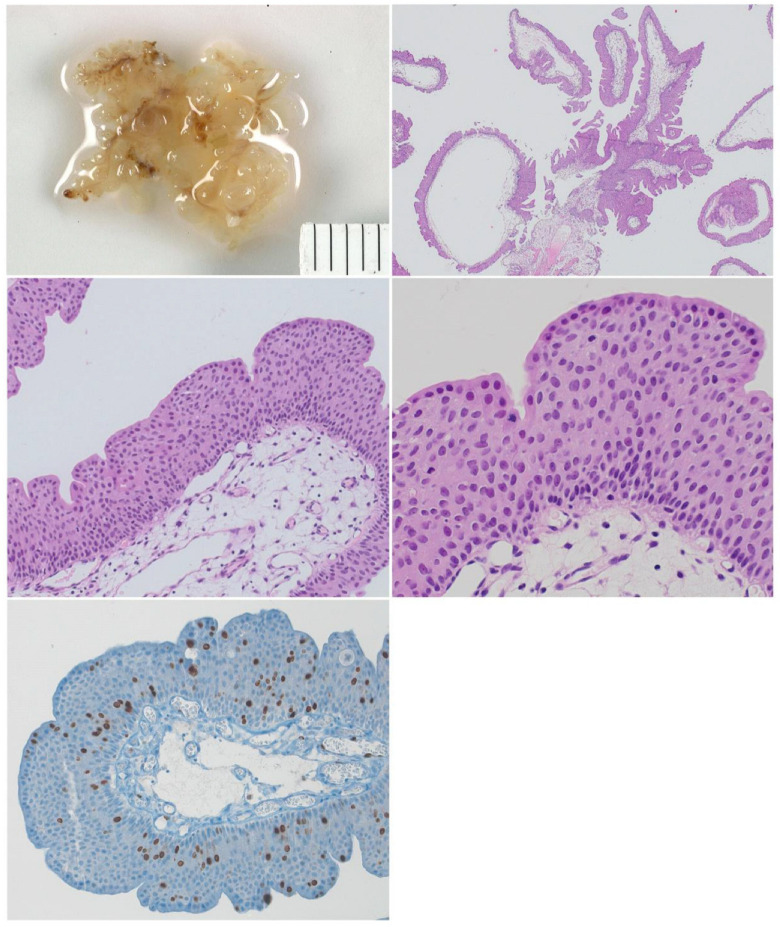
Papillary urothelial neoplasm of low malignant potential (PUNLMP). Macroscopic view (**left above**) and immunohistochemistry (HE).

Histopathology showed a papillary urothelial tumor with focal urothelial atypia. Immunohistochemistry revealed an increased proliferation up to the upper urothelial layers (Figure 3). In addition, we performed the UroVysion FISH test for chromosomal aberrations (3, 4, 17, and 9p21), and this test demonstrated deletion of 9p21. The tumor was classified as “papillary urothelial neoplasia of low malignant potential (PUNLMP)”.

The postoperative course was uneventful. Follow-up examinations included urine cytology, abdominal ultrasound, and cystoscopy including application of 5-ALA (fluorescence marker) after 3 months. Both examinations showed negative results. Urine cytology and abdominal ultrasound were repeated after 6 months and after 12 months. Afterward, annual follow-up included urine cytology and abdominal ultrasound for more than 7 years. No further routine cystoscopy was performed. Long-term follow-up for 120 months did not reveal any recurrence.

### 3.2. Patient No. 2

**Figure 4:**
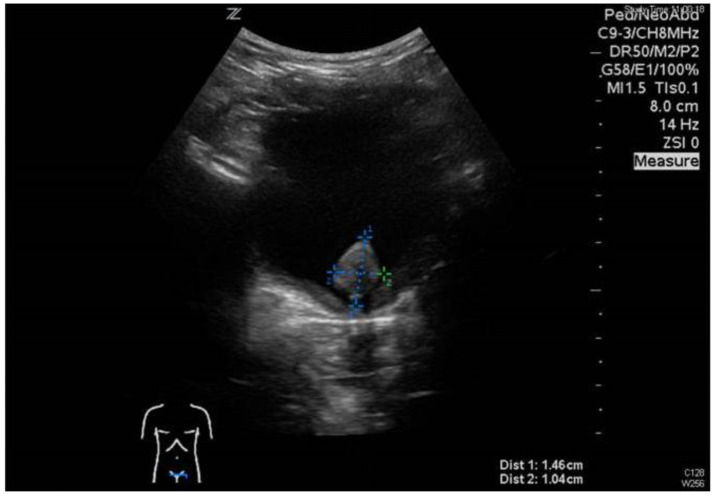
Solid mass located close to the bladder neck.

**Figure 5:**
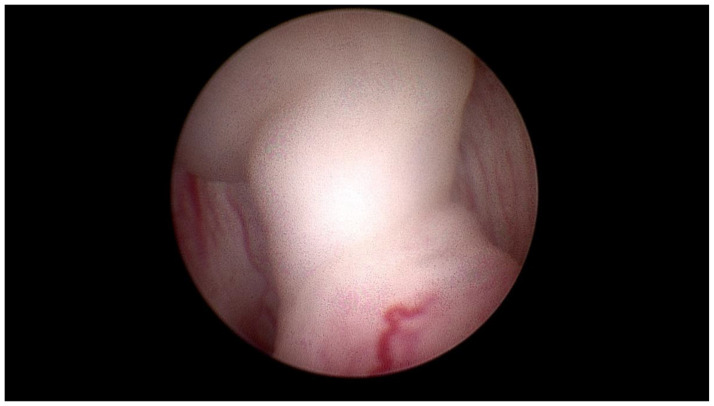
Cytoscopy showing a solid tumor arising from the colliculus seminalis reaching into the bladder neck.

A 4-year-old boy presented to our emergency room with two episodes of acute urinary retention within the last 48 h. The parents reported a history of several months of recurrent voiding disorders. Two years before the current admission, the boy presented for the first time with painful micturition and acute abdominal pain. At that time, clinical examination demonstrated mild abdominal tenderness, no other significant abnormality, no phimosis, and no evidence of trauma or manipulation at the external genitalia. Blood tests including coagulation status as well as urinalysis including bacteriology revealed no pathologic result. Abdominal and renal ultrasound revealed no pathologic finding. Afterward, the parents observed at least 10 additional episodes with similar symptoms within the last two years. Due to the recurrent painful episodes, the patient was scheduled to visit a pediatric urologist to discuss the indication for circumcision. At the current admission, clinical examination demonstrated a palpable, painful mass in the lower abdomen, but no other significant pathology and notably no phimosis. Again, blood tests including coagulation status as well as urinalysis including bacteriology revealed no pathologic result. Abdominal ultrasound showed a solid mass measuring 1.5cm in diameter, located close to the bladder neck (Figure 4). We performed transurethral cystoscopy which confirmed a solid tumor arising from the colliculus seminalis reaching into the bladder neck (Figure 5). The tumor was excised by complete transurethral resection.

**Figure 6:**
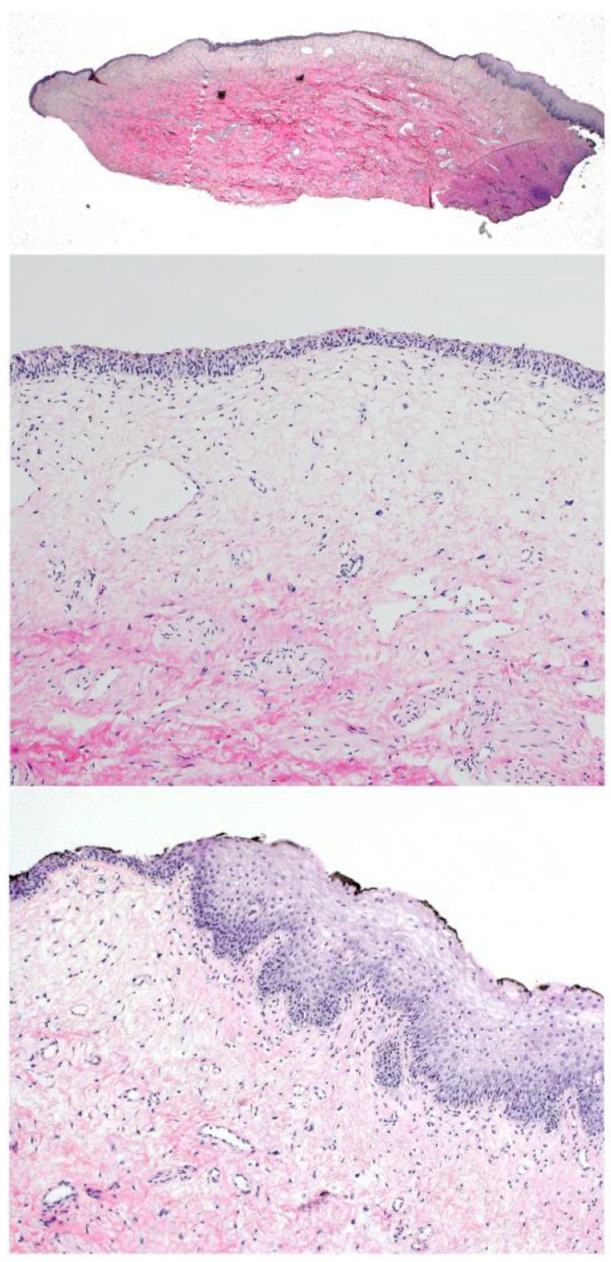
Fibroepithelial polyp (HE staining).

Histopathology showed a fibroepithelial polyp (Figure 6) covered by urothelial and squamous epithelial lining and a loose collagenous tissue core. There was no significant atypia and no major inflammation.

The postoperative course was uneventful. After 3 days, the transurethral catheter was removed. The patient micturated spontaneously without any pain, and an ultrasound showed no significant residual urine. Follow-up examinations demonstrated normal uroflowmetry and abdominal ultrasound after 3 months. Uroflowmetry and abdominal ultrasound were repeated after 6 months and after 12 months, again with negative results. Long-term follow-up to 48 months did not reveal any recurrence.

## 4. Discussion

Bladder tumors are still rare in the first decades of life [1,2,3,4,5,6,7,8,9,10,11,12,13,14,15,16]. We identified only five tumors in a retrospective analysis of our single-center database over 26 years. In our series, rhabdomyosarcoma (RMS) represents the most common malignant tumor. Besides RMS, two more tumors were diagnosed in our series: one papillary urothelial neoplasm of low malignant potential (PUNLMP) and one fibroepithelial polyp (FEP), a different entity each. These results are similar to the study of Alanee and Shukla [17]. 

The most important onset symptom was predominantly gross painless hematuria, followed by dysuria, isolated or recurrent. Painless hematuria as a directing symptom should never be underestimated. This observation is in accordance with the experience of nearly all other authors. Abdominal ultrasound was almost the first investigation and confirmed the diagnosis of a urothelial tumor in the great majority of patients. Transurethral resection is considered as the standard procedure to remove the tumor, performed by the majority of pediatric urologists during the same procedure. However, some authors perform transurethral biopsy followed by transurethral tumor resection in a second step [12,16]. 

Due to the different etiology of our two patients, we will discuss these two entities in detail below. In addition, we amend a review of the recent literature to analyze recommendations for diagnostic staging, treatment, and follow-up examinations as well as surveillance of patients suffering from urothelial tumors (excluding RMS) in the pediatric population.

## 5. Papillary Urothelial Neoplasia of Low Malignant Potential (PUNLMP)

Noninvasive papillary neoplasms (stage pTa) represent approx. 45% of all bladder tumors [18]. The prognosis of these lesions is largely defined by their pathologic grade. Several classification schemes have been advocated, with the last one corresponding to the 1998 World Health Organization/International Society of Urological Pathology (WHO/ISUP) classification system, which was modified in 2004 [2,19,20]. The 2004 WHO/ISUP system separates the noninvasive papillary neoplasms into four categories: urothelial papilloma, papillary urothelial neoplasm of low malignant potential (PUNLMP), low-grade urothelial carcinoma (LG-UC), and high-grade UC (HG-UC) [20,21]. Among these categories, PUNLMP is still considered as the most controversial of these pathological entities. A detailed description was reported previously [21]. PUNLMP is considered a neoplasm with a low biological risk of progression [18,21]. Therefore the term is applied to a lesion that is perceived to be less aggressive than an LG-UC but is yet not entirely benign and would necessitate clinical follow-up besides complete resection. Nevertheless, the 2004 WHO/ISUP classification defines a PUNLMP as a papillary urothelial tumor that grossly resembles an exophytic lesion but histologically shows increased cellular proliferation that exceeds the thickness of normal urothelium [2].

According to current understanding, bladder cancer is thought to arise from at least two different pathways: the *FGFR3*-associated pathway, and the *p53*-associated pathway [19]. *FGFR3* mutations are usually present in low-grade papillary carcinomas, whereas high-grade carcinomas are characterized by *p53* mutations [19]. FGFR3 mutations have been reported in urothelial and inverted papillomas but are rarely seen in pediatric patients [19,22].

Some molecular markers have been evaluated for the differential diagnosis of low-grade urothelial tumors, but there is no consensus regarding their usefulness or clinical implications. Immunohistochemical evidence of *p53* gene product overexpression is common in bladder cancer in young patients. Alteration of the *p53* gene is thought to be strongly associated with PUNLMP, although the pathogenetic mechanism is still unclear. Kalantari et al. showed that nuclear p53 protein in invasive high-grade transitional cell carcinoma (TCC) was slightly more frequent than that in noninvasive low-grade papillary TCC [23]. Only ten percent of the specimens with PUNLMP had nuclear p53 accumulation, while in low-grade and high-grade TCCs, 75% and 85% of the specimens were positive for p53 protein accumulation. They concluded that the overexpression of *p53* in papillary low-grade TCC and invasive high-grade TCC and the lack of *p53* expression in PUNLMP indicate that mutations of the *p53* gene are not usually associated with the development of urothelial neoplasms and may play a crucial role only in progression of PUNLMP to a higher-grade TCC [23].

Other genes and/or factors whose involvement is hypothesized are fibroblast growth factor-3 receptor (FGFR3) mutations as well as loss of heterozygosity (LOH) on chromosomes 3, 7, 9p, 9q, and 17p [24]. However, chromosomal abnormalities detected by UroVysion fluorescence in situ hybridization are sometimes present in patients above 19–20 years of age. This finding supports the proposed hypothesis that an age of 19–20 years separates distinct molecular pathways [24].

Urine cytology is a minimally invasive method of following adult patients. However, it must be kept in mind that the sensitivity decreases in well-differentiated tumors. Additionally, there is little experience reported with cytology in children [25,26]. Furthermore, there is a consensus that, at least in children, urine cytology alone does not represent a reliable marker to exclude a recurrence [25], which is confirmed by the majority of authors.

Definitive diagnosis of PUNLMP is performed by cystoscopy, which also allows evaluation of tumor extension, excision, or biopsy for histopathologic evaluation. As PUNLMP is generally solitary, non-muscle-invasive, and a low-malignancy tumor, its treatment is oriented to local excision only, and the prognosis is excellent in pediatric patients. Transurethral resection is considered as the “gold standard” procedure, performed by the majority of pediatric urologists during the same procedure. Recurrence or progression is uncommon in the young population [2]. In contrast, recurrences and progression to a higher grade urothelial lesion were observed in adult patients in 42% and 29% of patients, respectively [21]. However, our review identified two case reports including 9-year-old and 11-year-old male patients, respectively, suffering from relapsed disease after one year and more than 4 years, respectively [15,16].

A designation of PUNLMP avoids labeling a patient with “cancer” but does not equate these lesions with benign papillomas. The review by Fine et al. reclassifies the bladder lesions in a series of 23 patients with ages ranging from 4 to 20 years [2]. The authors found that there was no progression to carcinoma from the papillomas or PUNLMP. They were thus able to reclassify 43.5% of the patients in the series from carcinoma to PUNLMP and therefore exclude the diagnosis of “cancer” in these patients. With the publication of the 2004 WHO “blue book”, the nomenclature of urothelial neoplasms, and specifically papillary lesions, has been further refined to reflect biological behavior more accurately.

Due to the ongoing controversial histopathological nomenclature and the fact that PUNLMP represents still a rare entity in children, there is still no validated protocol or recommendation concerning preoperative staging as well as standardized follow-up examinations. Gross painless hematuria is the most relevant clinical syndrome. There is no doubt that abdominal ultrasound is the first investigation, performed by an experienced pediatric radiologist with a high specificity. However, there is no common consensus about any additional routine imaging before transurethral resection. In accordance with our oncology team, we performed preoperative computer tomography (CT) and MRI of the abdomen which showed no evidence of localized tumor spread and/or metastatic disease. Indication for thoracal CT, PET scan, and skeletal scintigraphy was discussed, but after intensive interdisciplinary discussion, we decided to refrain from performing these examinations. Costs and in particular risks of exposure to radiation have to be balanced against disease likelihood in pediatric patients.

Furthermore, there is still no standardized protocol for follow-up examinations available. The intensity of follow-up should be proportional to the risk of disease recurrence or progression. Patients with PUNLMP are often still followed for recurrences, in a manner comparable to those with a diagnosis of urothelial carcinoma. The best method of following these young patients after transurethral resection has been debated. Some authors advocate careful and extensive follow-up with interval cystoscopy as the primary modality. In contrast, others argue that ultrasound is extremely effective in identifying bladder tumors, and they recommend using ultrasound for initial diagnosis and disease surveillance. Adapting protocols from adult oncology, urine cytology is often a routine part of the follow-up program. Our first follow-up examinations after 3 months included urine cytology, abdominal ultrasound, and cystoscopy including application of 5-ALA, as recommended in the literature [27]. All examinations showed negative results. After one negative cystoscopy, only urine cytology and abdominal ultrasound were repeated after 6 months and after 12 months. Afterward, annual follow-up included abdominal ultrasound for more than 10 years. No further routine cystoscopy was performed. In our opinion, after one negative cystoscopy (including application of 5-ALA), the combination of clinical examination and abdominal ultrasound represents the most reliable protocol to follow these pediatric patients, which is in accordance with many other study results [12,13,14,15,16]. Maurizi et al. emphasized the result of a cystoscopy performed after 3 months as an important prognostic factor [15]. The relevance and usefulness of urine cytology as a follow-up parameter in pediatric patients with confirmed PUNLMP are discussed controversially and generally not recommended and accepted, respectively.

In our series, PUNLMP was less common than RMS, which is in contrast to the results of Alanee et al. [17]. Therefore, it would be useful to collect data from pediatric patients suffering from PUNLMP and establish an international central registry. The central registry would enable us to develop a standard operating protocol (SOP) for diagnostic work-up, treatment, and follow-up. Additionally, the results and consequently the relevance of genetic and molecular biologic aberrations could be evaluated systematically.

## 6. Fibroepithelial Polyp (FEP)

A fibroepithelial polyp (FEP) of the urinary bladder is a very rare benign congenital lesion in the pediatric population. An FEP is considered to be a non-neoplastic condition [3,6]. The most important onset symptom is predominantly dysuria and/or acute urinary retention, isolated or recurrent, as in our patient. Besides the patient’s history and clinical examination, ultrasound represents the first-line method to demonstrate the typical image of an irregular intravesical invading lesion, which may be associated with mild obstruction of the upper urinary tract. Diagnosis may be easily confirmed by cystoscopy, and transurethral resection is the treatment of choice. RMS of the bladder base or of the prostate as the most important differential diagnosis must be ruled out. Histological examination confirms the diagnosis of an FEP.

Since an FEP is considered as a benign lesion and no reports of recurrence and/or malignant transformation are known, recommendations for follow-up are different in comparison to PUNLMP. Follow-up examinations in our case included uroflowmetry and abdominal ultrasound after 3 months with negative results. Uroflowmetry and abdominal ultrasound were repeated after 6 months and after 12 months, again with negative results. After long-term follow-up for 48 months without any evidence of recurrence, scheduled follow-up was ceased, in accordance with recommendations of the literature [3,6].

## 7. Limitations

We report on a retrospective analysis from a single-center database, finally corroborating our hypothesis with only two case reports. However, with our data, we could confirm the experience mentioned in the recent literature reported from high-volume centers including a larger number of pediatric patients.

## 8. Conclusions

Both the review of the recent literature and the retrospective analysis of our surgical pathology record database confirm that bladder tumors in children are still rare and mostly consist of urothelial and mesenchymal neoplasms. In our series, RMS was the most common malignant bladder tumor in childhood. Urothelial lesions like PUNLMP as well as FEP are still rare in the pediatric population. In pediatric urothelial neoplasms, similar to those in adults, loss of 9p21 is implicated. The most important onset symptom is predominantly painless hematuria followed by dysuria, isolated or recurrent. Abdominal ultrasound is the first-line method to confirm the diagnosis of a urothelial tumor, and transurethral resection is the standard procedure to remove the tumor. 

General treatment protocols including recommendations for staging, tumor markers, and follow-up examinations are still not yet available for this tumor entity in the pediatric population. Implementation of a central international database would enable us to develop a standard operating procedure for diagnosis, treatment, and follow-up of these patients.

## Data Availability

Due to privacy and ethical restrictions, our data is not available.
